# Plasma-based microRNA biomarkers for depression in Romanian patients: preliminary findings

**DOI:** 10.1192/j.eurpsy.2025.1535

**Published:** 2025-08-26

**Authors:** I.-C. Matei, E. Milanesi, M. Dobre

**Affiliations:** 1 Clinica Nutrimed; 2 Institutul National de Patologie, Bucharest, Romania

## Abstract

**Introduction:**

Depressive disorders are one of the most disabling mental illnesses with a significant impact on society. In Romania the mean annual total costs per depression patient are EUR 5553, a much higher figure than the mean annual total costs per patient ranged worldwide.

Even though their roles in depression have not been elucidated, a plethora of potential biomarkers in multiple body fluids for the early diagnosis of depression has been suggested. Blood circulating microRNAs (miRNAs) are potential biomarkers for several human diseases, including psychiatric disorders. Different studies have shown that micro RNAs are involved in a series of pathophysiological processes and could be useful markers for diagnosis and prognosis of depression.

**Objectives:**

This preliminary case-control study was designed to identify putative blood circulating miRNAs associated with the diagnosis of depression in Romanian patients.

**Methods:**

In this study, 20 patients with depression and 24 non-depressed controls were enrolled. All the individuals have been interviewed and screened using the following scales: the Hamilton Anxiety Scale (HAM-A), the Becks Depression Inventory (BDI) and the Perceived Stress Scale (PSS). The expression of 179 miRNAs in plasma have been evaluated by qRT- PCR. The difference in the expression of miRNAs between the two groups, as well as the correlations with the scores of the scales have been analyzed.

**Results:**

A panel of 28 miRNAs was identified as differentially expressed between patients and controls. Only miRNAs showing a -2>FC>2.0 and p<0.05 have been considered significant. Seven miRNAs (miR-143-3p, miR-331-3p, let-7f-5, miR-502-3p, miR-145-5p, miR-7-1-3p, miR- 29a-3p) were found up-regulated in the depression group, while 21 miRNAs (miR-885-5p, miR- 425-3p, miR-32-5p, miR-23b-3p, miR-590-5p, miR-30a-5p, miR-132-3p, miR-376a-3p, miR-223-5p, miR-133b, miR-142-5p, miR-92b-3p, miR-140-3p, miR-16-2-3p, miR-28-3p, miR-27a-3p, miR-15b-3p, miR-106b-3p, miR-877-5p, miR-30e-3p, miR-140-5p) showed a down-regulation in the group of patients compared to the controls. Some of the significant correlations between miRNA expression and the scale scores are reported: a positive correlation between let-7f-5 and miR-7-1-3p with the BDI score (p=0.003 r=0.526, and p=0.008 r=0.477, respectively) and a negative correlation between miR-425-3p with BDI (p=0.002 r=-0.502) were found.

**Conclusions:**

The results reported in this communication represent preliminary findings. Due to the nature and heterogeneity of depression, the number of patients and controls in the two cohorts will be enlarged to correlate these miRNAs with other patient features.

**Disclosure of Interest:**

None Declared

